# Mesenchymal stem cell spheroids exhibit enhanced *in-vitro* and *in-vivo* osteoregenerative potential

**DOI:** 10.1186/s12896-014-0105-9

**Published:** 2014-12-06

**Authors:** Yuichiro Yamaguchi, Jun Ohno, Ayako Sato, Hirofumi Kido, Tadao Fukushima

**Affiliations:** Department of Oral Rehabilitation, Section of Oral Implantology, Fukuoka Dental College, 2-15-1 Tamura, Sawara-ku, Fukuoka, 814-0193 Japan; Department of Morphological Biology, Section of Pathology, Fukuoka Dental College, 2-15-1 Tamura, Sawara-ku, Fukuoka, 814-0193 Japan; Center for Regenerative Medicine, Fukuoka Dental College, 2-15-1 Tamura, Sawara-ku, Fukuoka, 814-0193 Japan

**Keywords:** Spheroid, Mesenchymal stem cell, Osteogenesis, Rat calvarial defect, Bone regeneration

## Abstract

**Background:**

Mesenchymal stem cells (MSCs) are a favored cell source for regenerative medicine because of their multilinage potential. However, the conventional monolayer technique used to culture MSCs, inadequately overcomes their low differentiation capacity. Culture of MSCs in multicellular spheroids, more accurately mimics the *in-vivo* microenvironment; thus, resolving this problem. In this study, we assessed whether the osteoregenerative potential of MSC spheroids is greater than that of monolayer MSCs.

**Results:**

MSC spheroids were generated from rat MSCs (rMSCs) using low-binding plates. Real-time reverse transcription-polymerase chain reaction and immunocytochemical analysis indicated that osteogenic properties were accelerated in MSC spheroids compared with monolayer rMSCs when treated with an osteoblast-inducer reagent for 7 days. Moreover, increased calcium deposition was visualized in MSC spheroids using Alizarin red staining. In a rat calvarial defect model, micro-computed tomography and histological assays showed that MSC spheroid-engrafted defects experienced enhanced bone regeneration.

**Conclusions:**

Our *in-vitro* and *in-vivo* results reveal that MSCs in the spheroid culture exhibit enhanced osteoregenerative efficiency compared with monolayer MSCs.

## Background

Adult stem cells are widely used as a cell source for regenerative medicine because of their multilineage potential. These cells are easily harvested from bone marrow [1,2], adipose tissue [3,4], and other sites [5-9]. Bone marrow contains hematopoietic stem cells, mesenchymal stem/stromal cells (MSCs), and multipotent adult progenitor cells [10-12]. MSCs are capable of self-renewal and differentiation into several mesenchymal lineages *in-vitro* and *in-vivo*, including bone, fat, cartilage, and skeletal muscles [12,13]. These are considered suitable for use in tissue regeneration. As culture in the presence of osteogenic supplements facilitates MSCs to undergo differentiation into the osteogenic phenotypes, MSCs have been utilized as a cell source for osteogenic tissue regeneration [10,11]. Bone marrow-derived MSCs have great potential for bone regeneration in future clinical applications.

MSCs are commonly cultured as a two-dimensional (2D) monolayer using conventional tissue-culture techniques. These 2D-monolayer techniques inadequately reproduce the *in-vivo* microenvironment of stem cells, established by extrinsic and intrinsic cellular signals and have a profound influence on their biological functions [14]. Culturing multipotent MSCs in a 2D adherent monolayer can alter their normal physiological behavior, resulting in the loss of replicative ability, colony-forming efficiency, and the differentiation capabilities over time [15,16]. Replication of this complex *in-vivo* microenvironment *in-vitro* requires highly sophisticated cell-culture systems.

To mimic the *in-vivo* microenvironment more accurately *in-vitro*, various three-dimensional (3D) culture systems have been developed. Spheroids, spherical clusters of cells formed by self-assembly, comprise one of the best models for the 3D culture [17,18]. Our understanding of the spheroid cell biology mainly derives from the *in-vitro* culture of cancer cell lines. Many studies have highlighted significant differences between 2D and 3D cultures, with the latter better reflecting the *in-vivo* microenvironment in terms of cellular heterogeneity, nutrient and oxygen gradients, cell-cell interactions, matrix deposition, and gene expression profiles [19-24]. Previous studies have reported several methods of generating 3D MSC spheroids. Many of these methods involve the use of a cell-suspension system or nonadherent surface to induce the spheroid formation [25-27]. In general, these MSC spheroids possess a greater differentiation capacity.

In this study, we examined the osteogenerative potential of the spheroids of rat MSCs (rMSCs) isolated from bone marrow compared with monolayer rMSCs in both *in-vitro* assays and a rat calvarial defect model, to elucidate whether MSC spheroids exhibit enhanced bone regeneration.

## Results

### Mesenchymal stem cell spheroid formation

**Figure 1:**
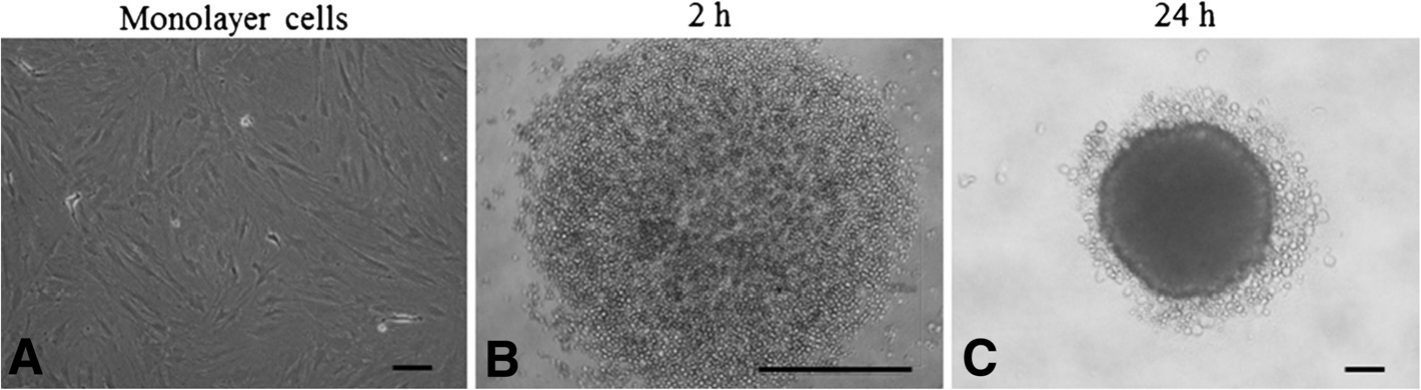
**Mesenchymal stem cell spheroid, generated from rat mesenchymal stem cell lines. (A)** Parental cell line grown permanently as a monolayer culture. **(B)** and **(C)** The generation of mesenchymal stem cell (MSC) spheroids. Cellular aggregation of MSCs was observed 2 h after a microplate culture **(B)**. A cell aggregation formed a compact spheroid at 24 h **(C)**. Scale bars = 100 μm (a and c) and 1 mm **(B)**.

First, we examined the generation of MSC spheroids from rMSCs (Figure 1A) at a density of 1 × 10^4^ cells/well using round-bottom, low-binding plates. Culture of rMSCs in the low-binding plates generated a single spheroid per well. At 2 h after seeding 100 μl of rMSC cell suspension into the plates, cells spontaneously aggregated in the medium (Figure 1B). Cell aggregates formed compact multicellular spheroids 24 h after suspension culture (Figure 1C).

**Figure 2:**
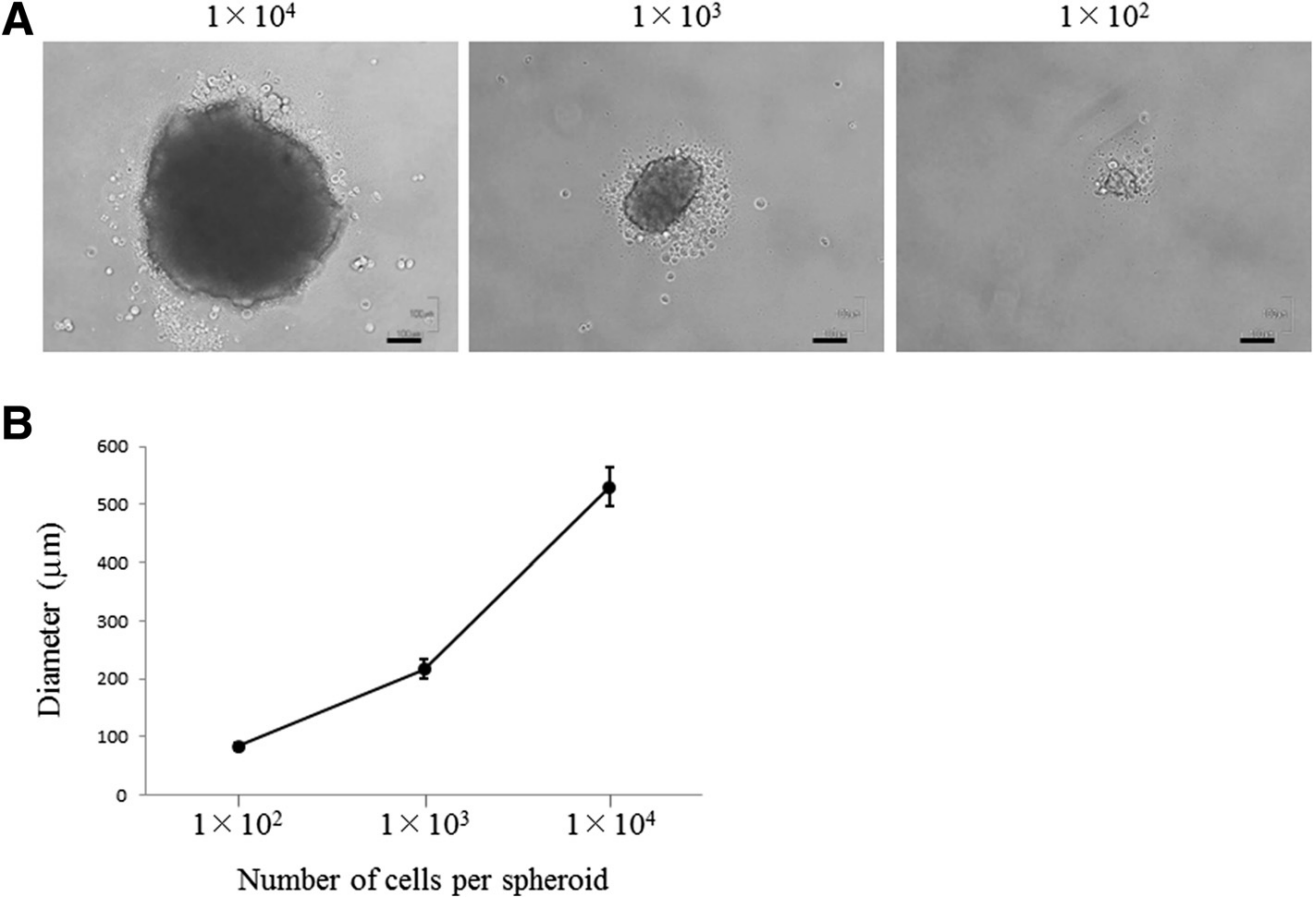
**Effect of seeding densities on a spheroid formation. (A)** Representative images of mesenchymal stem cell (MSC) spheroids grown on low-binding plates at 24 h after implantation. Scale bar = 100 μm. **(B)** The diameter of MSC spheroids increased linearly with increasing cell number.

Furthermore, we investigated the effects of seeding density (10,000, 1,000, or 100 per well) on the spheroid formation. After the spheroids had been allowed to aggregate for 24 h, the spheroid diameter was quantified via bright field microscopy (*n* = 5 per seeding density). The spheroid diameter increased with increasing cell number, ranging from 83.2 ± 6 μm (6 micrometer) for the 100-cell spheroid to 530 ± 34.3 μm (34.3 micrometer) for the 10,000-cell spheroid (Figure 2A and B). These results indicate that the spheroid size can be controlled by cell seeding density.

### Increased expression of osteogenic genes in mesenchymal stem cell spheroids treated with an osteoblast-inducer reagent

**Figure 3:**
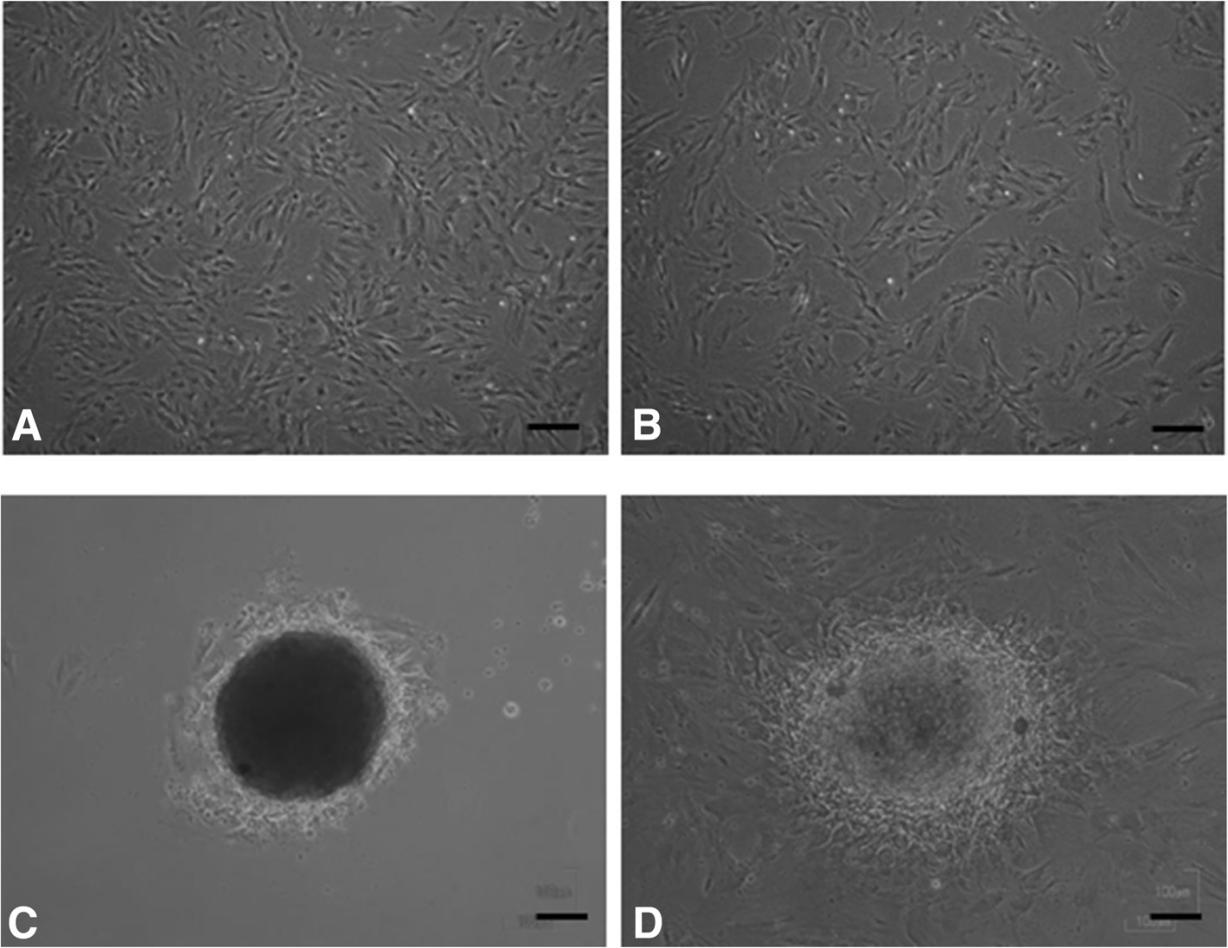
**Cytological images of differentiated monolayer mesenchymal stem cells (MSC) and MSC spheroids.** No cytological changes were observed inboth monolayer MSCs and MSC spheroids cultured with an osteoblast-induced reagent. Untreated monolater MSCs **(A)**, treated monolayer MSCs **(B)**, untreated MSC spheroids **(C)**, and treated MSC spheroids **(D)**. Bars = 100 μm.

**Figure 4:**
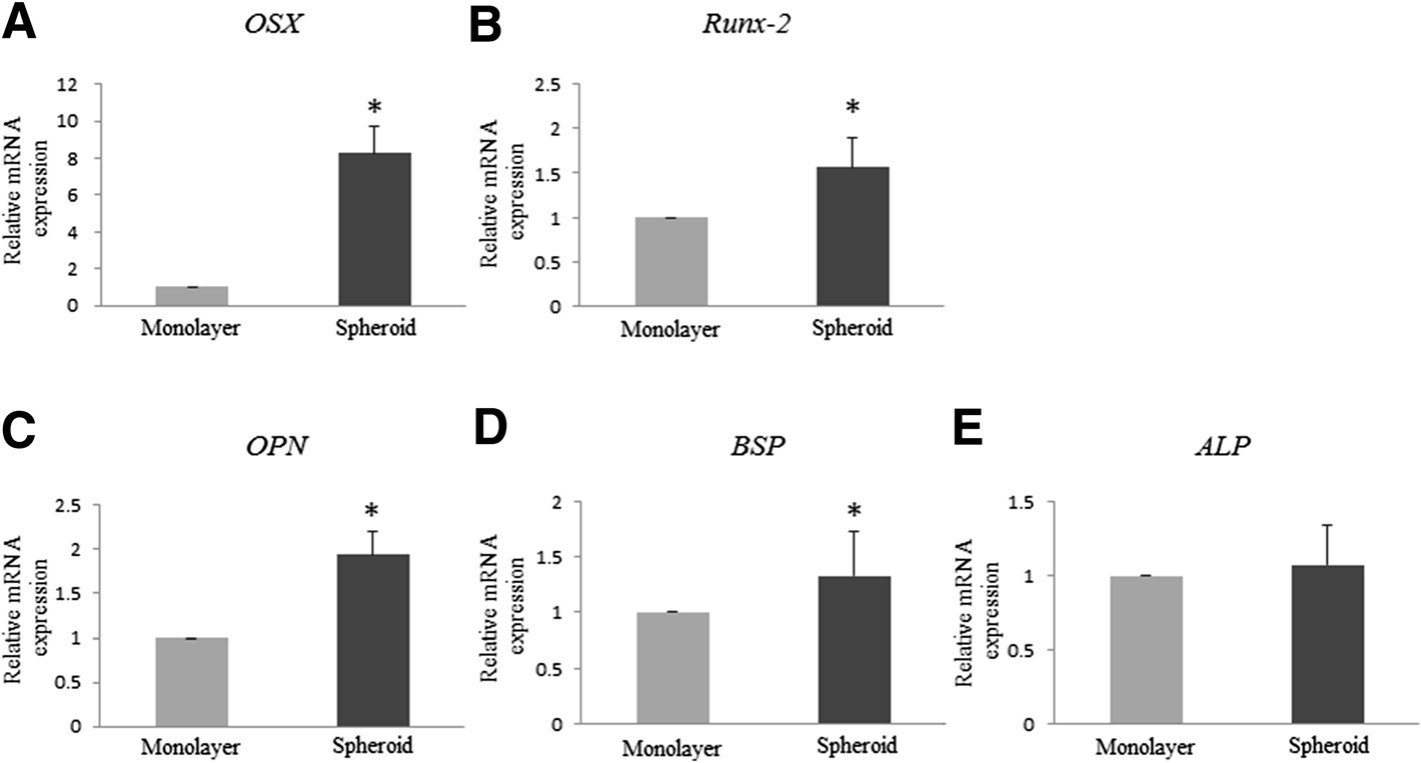
**Expression of osteogenic marker genes in both mesenchymal stem cell spheroids and monolayer rat mesenchymal stem cells.** Quantitative reverse transcription-polymerase chain reaction results for *runt-related transcription factor-2*, *osterix*, *osteopontin*, and *bone sialoprotein*, and *alkaline phosphatase* mRNA expression in mesenchymal stem cell (MSC) spheroids (black column) and monolayer rat MSCs (rMSCs; gray column) treated with an osteoblast-inducer reagent (OIR). Results are expressed as fold-increases in mRNA expression (normalized to that of *glyceraldehyde 3-phosphate dehydrogenase* mRNA) and compared with results for monolayer rMSCs treated with an OIR on Day 7. mRNA expression levels of osteogenic genes, other than alkaline phosphatase (ALP) **(E)**, in MSC spheroids were increased, compared with those of monolayer MSCs **(A-D)**. Vertical lines represent the mean ± standard deviation of three independent experiments, each performed in triplicate. *Significantly different at p < 0.05 compared with monolayer rMSCs. *OSX*, osterix; *RUNX-2*, runt-related transcription factor-2; *OPN*, osteopontin; *BSP*, bone sialoprotein; *ALP*, alkaline phosphatase.

After treatment with an osteoblast-inducer reagent (OIR) for 7 days, no cytological changes were observed in both monolayer MSCs and MSC spheroids (Figure 3). The spheroid diameter slightly decreased for 7-day treatment with OIR, ranging from 327.8 ± 17.8 μm on day 0 to 290.3 ± 33.8 μm on day 7. To investigate whether osteogenic genes are induced in MSC spheroids treated with an OIR, the relative mRNA expression levels of *runt-related transcription factor-2* (*RUNX-2)*, *osterix* (*OSX)*, *osteopontin* (*OPN)*, and *bone sialoprotein* (*BSP)* mRNA expression were analyzed using quantitative reverse transcription-polymerase chain reaction (RT-PCR). Compared with monolayer rMSCs treated with an OIR, the relative mRNA expression levels of *OSX* (normalized to that of *glyceraldehyde 3-phosphate dehydrogenase* [G3PDH] mRNA) increased by 8.27-fold (Figure 4A). Similarly, the mRNA expression levels of *RUNX-2, OPN,* and *BSP* increased by 1.57-, 1.94-, and 1.33-fold, respectively (Figure 4B,C, and D). In contrast, *alkaline phosphatase* (ALP) mRNA expression remains unchanged in both MSC spheroids and monolayer rMSCs (Figure 4E). The levels of expression of these osteogenic genes were significantly upregulated in MSC spheroids compared with monolayer rMSCs.

### Immunocytochemical expression of osterix in differentiated mesenchymal stem cell spheroids

**Figure 5:**
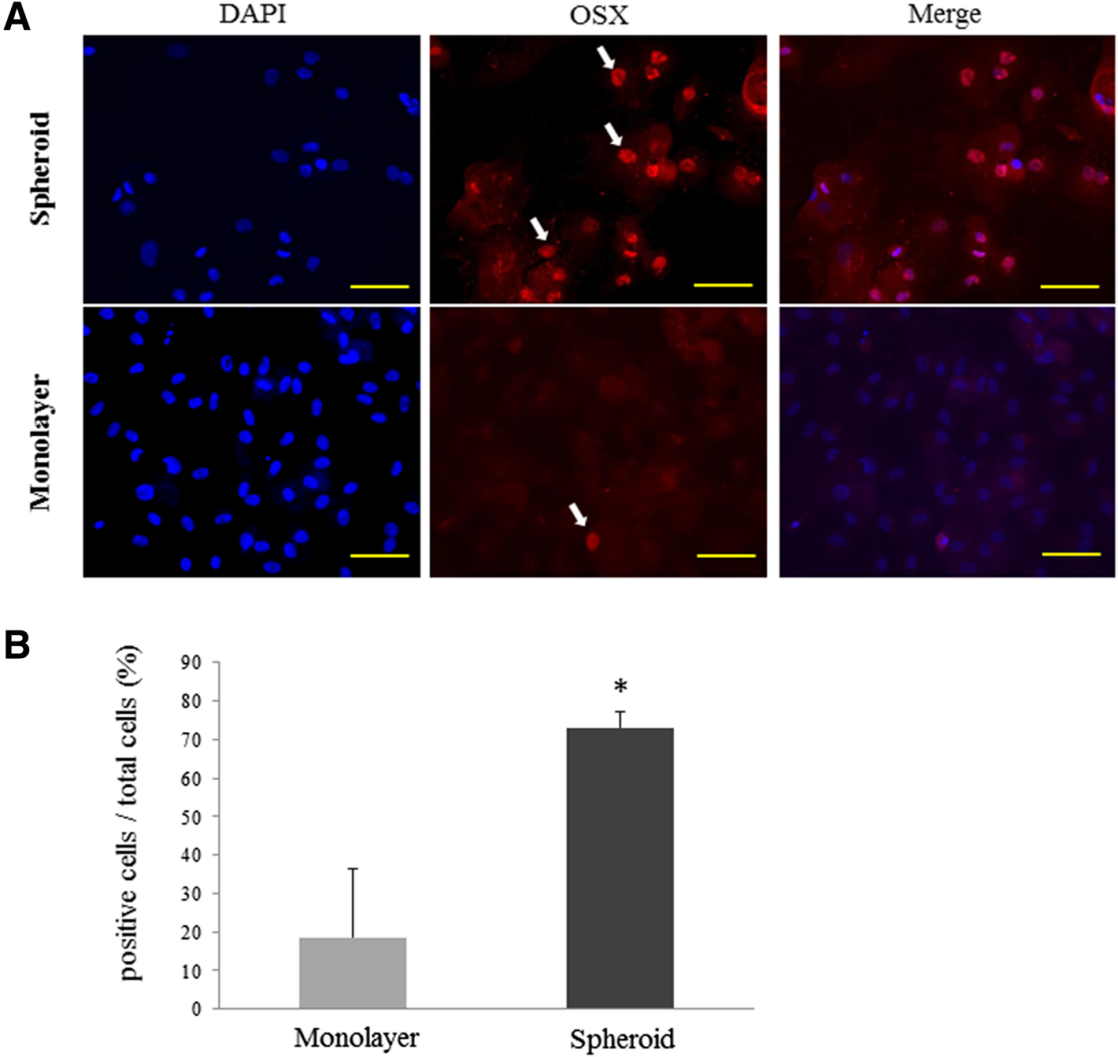
**Immunocytochemical analysis of osterix expression in mesenchymal stem cell spheroids treated with an osteoblast-inducer reagent for 7 days. (A)** Compared with monolayer mesenchymal stem cell **(**MSC; lower), positive cells (red) with anti- osterix antibody were increased in MSC spheroids (upper). 4′,6-diamidino-2-phenylindole (DAPI) was used to label nuclei (blue). Arrows, positive reaction in nuclei. Scale bars = 100 μm. **(B)** Significantly increased percentage of osterix positive cells was observed in MSC spheroids when compared to monolayer MSCs (*, p < 0.01).

Furthermore, we performed immunocytochemical analysis to examine the protein expression of the osteogenic marker OSX in spheroid and monolayer rMSCs, cultured with medium containing an OIR (Figure 5). MSC spheroids exhibited red-colored nuclear fluorescence (Figure 5A, upper). In contrast, monolayer MSCs showed no staining in the nuclei, and only faint staining was evident in the cytoplasm (Figure 5A, lower). A significant increased percentage of total osterix positive cells was observed in MSC spheroids as compared to monolayer MSCs [total: 73.0 ± 4.0 vs 18.5 ± 1.8 (p < 0.01)] (Figure 5B). These results reveal that OSX expression is induced in the nuclei of spheroid MSCs treated with an OIR for 7 days.

### Increased alkaline phosphatase staining and induction of calcified deposits in mesenchymal stem cell spheroids treated with an osteoblast-inducer reagent

**Figure 6:**
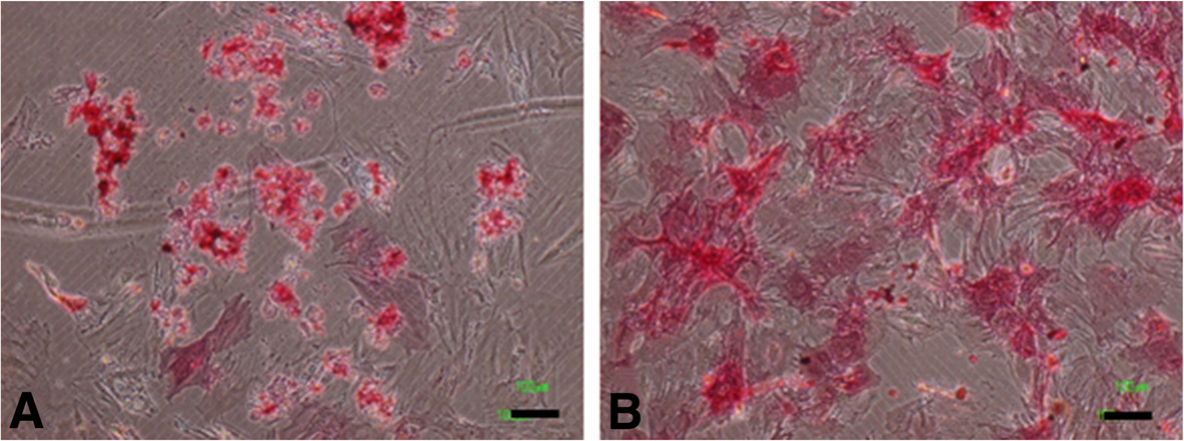
**Alkaline phosphatase staining in mesenchymal stem cell spheroids and monolayer rat mesenchymal stem cells.** Alkaline phosphatase staining was observed in both mesenchymal stem cell (MSC) spheroids **(A)** and monolayer rat MSCs **(B)**. Scale bar = 100 μm.

ALP staining intensity and Alizarin red (AR) deposits are well-defined cytochemical markers of osteogenesis and are considered to reflect the differentiation into osteoblasts [28,29]. We compared the staining intensity for ALP and AR in MSC spheroids and monolayer rMSCs, cultured with an OIR for 7 days. ALP staining intensity was evident in both MSC spheroids (Figure 6A) and monolayer rMSCs (Figure 6B) cultured with an OIR for 7 days. These results indicate that both spheroids and monolayer cells can differentiate into progenitors for bone formation after an OIR stimulation.

**Figure 7:**
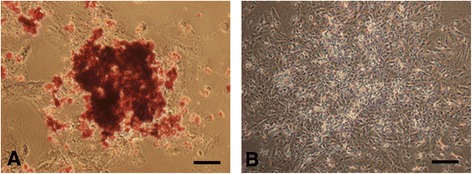
**Mineralization of mesenchymal stem cell spheroids cultured with an osteoblast-inducer reagent.** Mesenchymal stem cell (MSC) spheroids **(A)** and monolayer rat MSCs **(B)** were incubated for 7 days with an osteoblast-inducer reagent (OIR). Matrix mineralization was visualized in OIR-cultured MSC spheroids using Alizarin red staining. Scale bar = 100 μm.

AR staining of calcified deposits was used to examine the mineralization of the extracellular matrix. We investigated whether MSC spheroids cultured with an OIR for 7 days, exhibit a late period of matrix maturation. The centeral areas of MSC spheroids (Figure 7A) appeared red, whereas no spots of AR staining were evident in monolayer rMSCs (Figure 7B). These results indicate that MSC spheroids treated with an OIR induce more calcification than monolayer rMSCs.

### Mesenchymal stem cell spheroids enhance the healing of rat calvarial defects

**Figure 8:**
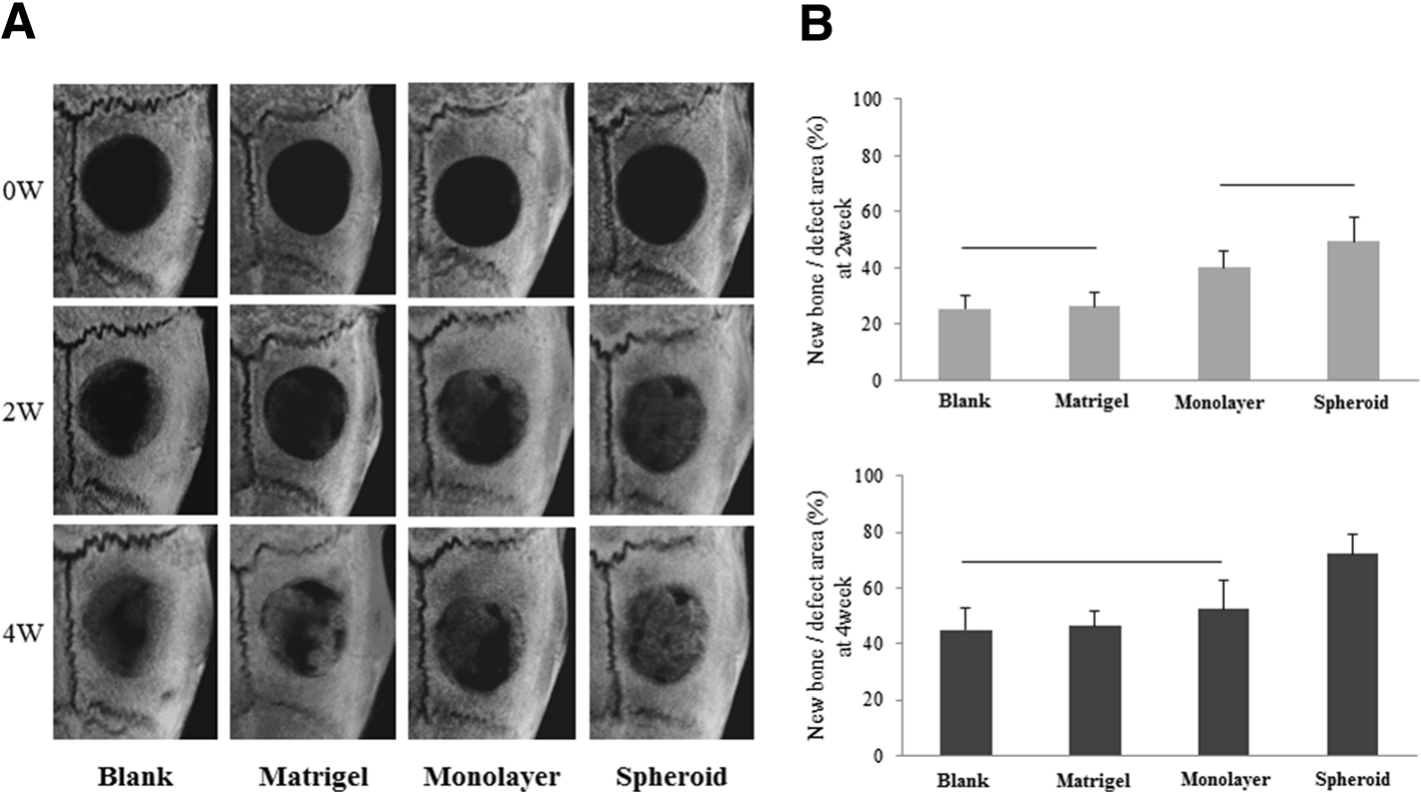
**Healing of rat calvarial defects assessed by micro-computed tomography. (A)** Micro-computed tomography (micro-CT) images of the calvaria, 0, 2, and 4 weeks after implantation of the indicated materials. Newly regenerated bone in the blank, Matrigel™ alone, monolayer rat mesenchymal stem cells (MSCs)/ Matrigel™, and MSC spheroids/ Matrigel™ groups were evaluated. In the blank group, the defect was left unfilled. **(B)** Quantitative results for micro-CT analysis. Healing of defects was shown as the fraction of new bone with respect to the total defect area as quantified by micro-CT analysis (n = 5 per group). There were statistical differences between the area of new bone formed in the MSC spheroids group and that in the other groups (p < 0.05). There are no significant difference in groups of gray columns jointed to horizontal bar.

To determine whether MSC spheroids enhance bone regeneration, we implanted MSC spheroids/Matrigel™ (BD Biosciences, San Jose, CA, USA), monolayer rMSCs/Matrigel™, Matrigel™ alone, or nothing (blank) into rat calvarial bone defects. The area of regenerated bone was investigated by micro-computed tomography (micro-CT) scanning (Figure 8A). At 2 and 4 weeks after implantation, bone defect areas were still evident in the blank, Matrigel™ alone, and monolayer rMSC/Matrigel™ groups. Conversely, the defect areas were small in the MSC spheroid/Matrigel™ group because they were filled with regenerated bones.

We quantified these micro-CT findings to yield the percentage of defect healing by quantifying the pixels in these defects. These results are summarized in Figure 7B. MSC spheroid/ Matrigel™ -implanted rats (49.4 ± 8.7% at 2 weeks; 72.2 ± 7.0% at 4 weeks) showed higher percentages of new bones at 4 weeks compared with rats in the other groups. The area of regenerated bones in rats in the other three groups was less than 55% (blank: 25.6 ± 4.9% and 45.0% ± 8.1%, Matrigel™ alone: 26.3 ± 5.0% and 46.2 ± 5.6%, monolayer rMSC/ Matrigel™: 39.9 ± 6.0% and 52.3 ± 10.2% at 2 and 4 weeks, respectively]. Thus, the MSC spheroids group showed higher bone regeneration in the peripheral area surrounding the defect compared with other groups.

**Figure 9:**
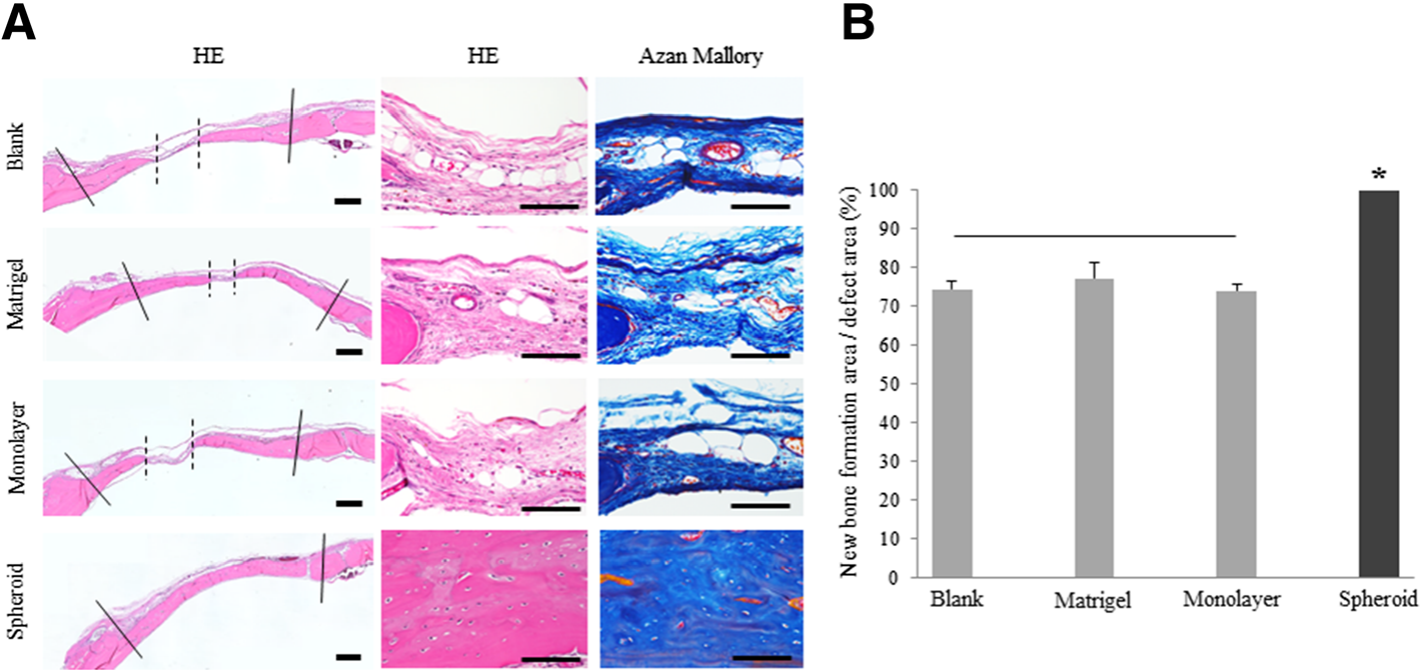
**Histological analysis of regenerated bone after implantation. (A)** Hematoxylin and eosin (HE) and Azan Mallory staining of calvarial tissue sections was conducted 4 weeks after implantation. Newly regenerated bone in the blank, Matrigel™ alone, monolayer rat mesenchymal stem cells (MSCs)/Matrigel™, and MSC spheroids/Matrigel™ groups were evaluated histologically. The left and middle columns showed HE staining at low and high magnification, respectively. Solid lines indicate the edges of the host bone. Dotted lines indicate the defect filled with the connective tissue. In MSC spheroids/Matrigel™ group, the defect were filled with newly regenerated bone. In the right column showing Azan Mallory staining, the defects of the blank, Matrigel™ alone, monolayer rat mesenchymal stem cells (MSCs)/Matrigel™ groups were filled with mature collagenous fibers. Scale bar in the left column = 1 mm. Scale bars in the middle and right columns = 100 μm. **(B)** Percentages of newly regenerated bone in relation to the size of the original defect evaluated histomorphometricly 4 weeks after implantation. Replacement of newly bone in MSC spheroids/ Matrigel™ group was greater than that in other groups. *, significantly different at p < 0.05 compared with other groups. There are no significant difference in groups of gray columns jointed to horizontal bar.

Histological analysis also revealed that the bones were well generated in the MSC spheroids group compared with the other groups. At 4 weeks after implantation, the bone defects were almost replaced by regenerated bone in the MSC spheroids group. In the other groups, some newly regenerated bones were evident, but considerable areas of the bone defects remained filled with fibrous connective tissue. No inflammatory changes were observed in any group (left and middle columns in Figure 9A). Azan Mallory staining showed that areas of the bone defects were filled with mature collagenous fibers in the blank, Matrigel™ alone, monolayer MSC/Matrigel™ groups (right column in Figure 9A). A histomorphometric analysis represented that 74.4 ± 1.9% and 77.1 ± 4.1% of the bone defects were filled with new bone in the blank and Matrigel™ alone groups, respectively. In the monolayer MSC/Matrigel™ group, 73.9 ± 1.8% of the defects were filled with newly regenerated bone (Figure 9B).

## Discussion

The spheroid culture system has some advantages over the standard monolayer culture. The methods of the spheroid culture, allows the cells to adapt to their native morphology, facilitating greater cell-cell contacts and interactions between the cells and the extracellular matrix. In this study, we present two lines of evidence to support the concept that MSC spheroids exhibit enhanced osteogenic potential compared with monolayer rMSCs cultured, using conventional techniques. First, *in-vitro* assays confirmed that MSC spheroids upregulate osteogenic genes and proteins and also exhibit increased calcium deposition. Second, the implantation of MSC spheroids *in vivo* enhanced bone regeneration in rat calvarial defects.

A standardized microplate method used in this study generated a single MSC spheroid in each well. The round-bottom, low-binding plates had the following desirable characteristics: (i) a single spheroid per well, centered for ease of optical imaging; (ii) high reproducibility; (iii) simple harvesting for further analysis [30]. It was possible to form spheroids by altering the initial seeding density of rMSCs. Spheroids size must be important because of limitations in the length of nutrient transport by diffusion. Curcio et al. reported that the spheroid radii greater than 200 μm rendered cells in their core vulnerable to hypoxia and cell death [31]. In both *in-vitro* and *in-vivo* experiments, we used the spheroids containing approximately 10,000 cells with a mean radius of approximately 200 μm. Conversely, delivery of a substantial number of cells is required for cell-based therapies to drive tissue formation, and larger spheroids facilitate the transplantation of fewer aggregates.

The results of our *in-vitro* assays indicate that osteogenic properties are accelerated in MSC spheroids treated with an OIR. We compared changes the osteogenic gene expression profiles of 3D MSC spheroids vs. 2D monolayer rMSCs by quantitative RT-PCR. Expression of the *RUNX-2*, *OSX*, *OPN*, and *BSP* genes were upregulated in MSC spheroids treated with an OIR compared with monolayer rMSCs. The process of osteoblastic differentiation is regulated by multiple factors and signaling pathways. Among the upregulated genes, *RUNX-2*, also called *core-binding factor A1*, and *OSX* are often referred to as the “master switch” of osteogenic differentiation [32,33]. It is established that bone genes are crucial for the generation of a mineralized tissue, and are usually analyzed during the early phases of osteogenic differentiation. MSC spheroids treated with an OIR for 7 days appear to begin toward the osteogenic differentiation. Our immunocytochemical findings showing OSX expression in MSC spheroids support this speculation.

Interestingly, our PCR results also showed an upregulation of the intermediate to late markers of osteogenesis, *OPN* and *BSP. OPN* is a noncollagenous, secreted glycosylation phosphoprotein expressed during both the early and the intermediate stages of osteogenesis [34-36]. *BSP* is also a noncollagenous, acidic phosphoprotein normally expressed in mineralized tissues such as bone and dentin [37]. Upregulation of these genes prompts us to suggest that they may serve as a matrix-associated signal directly promoting osteogenic differentiation and resulting in the increased production of a mineralized matrix in MSC spheroids. Using AR staining, we found increased calcium deposition in MSC spheroids treated with an OIR for 7 days. It is difficult to explain why the mixtures of various differentiation phases are contained within MSC spheroids. We believe that the multicellular spheroids consist of cells at various stages of osteogenic differentiation. Future work will address how osteogenic differentiation is regulated in the cells consisting of MSC spheroids.

In our *in-vitro* experiments, there were no differences in *ALP* gene expression and ALP activity between MSC spheroids and monolayer rMSCs. ALP is another important marker, as an effector protein responsible for osteoprogenitor markers and mineralization of the extracellular matrix during osteogenic differentiation [38]. In this study, we found that the expression levels of *ALP* mRNA in monolayer rMSCs treated with an OIR for 7 days was identical to that in MSC spheroids. ALP staining was of identical intensity between monolayer rMSCs and MSC spheroids treated with an OIR for 7 days, but no AR deposits were observed in monolayer rMSCs. These results suggest that monolayer rMSCs can differentiate into a progenitor phase without the initiation of mineralization. Therefore, MSC spheroids treated with with an OIR exhibit enhanced the differentiation capabililties compared with monolayer rMSCs.

Micro-CT analysis of MSC spheroid-engrafted calvarial defects in rats indicates that these spheroids have a dramatic effect on bone regeneration. Histological findings complemented these micro-CT results, which showed that a bone bridge had almost covered the defect in rats in the MSC spheroids group, although the fibrous connective tissue remained in the defect in rats in the other groups. Based on the histological findings of engrafted defects in the control groups, we suggest that the bone healing process consistes of the extensions of new bone from the edges of the defect. This healing process is supported by our previous report [29]. Our *in-vitro* and *in-vivo* results reveal that MSCs in the spheroid culture exhibit enhanced osteogenic efficiency compared with those in the monolayer culture. However, the detailed mechanisms that enhanced *in-vitro* osteogenesis and *in-vivo* bone regeneration by which MSC spheroids occur remain unclear. Future studies will address the detailed effects of MSC spheroids on microenvironments during the induction of osteogeneis in bone defects.

## Conclusion

Our *in-vitro* experiments on osteogenic induction showed that MSC spheroids possessed enhanced osteogenic potential compared with monolayer rMSCs. Moreover, we demonstrated that engrafted MSC spheroids induced efficient bone regeneration in calvarial defects in rats. Based on these results, we conclude that MSCs in the spheroid culture exhibit enhanced the osteogenic capabilities.

## Methods

### Cell lines

Bone marrow-derived rMSCs, isolated from Fischer 344 rats, were purchased from Lonza Group AG (Basel, Switzerland) and expanded according to the established protocols. Briefly, cryopreserved rMSCs were thawed and plated onto a 10-cm tissue-culture dish in 10 ml Dulbecco’s modified Eagle’s medium (DMEM; Wako Pure Chemical Industiries,Ltd., Osaka, Japan) supplemented with 10% fetal bovine serum (FBS; Sigma-Aldrich Corporation, St. Louis, MO, USA), and 1% (v/v) penicillin/ streptomycin (PS; Invitrogen/GIBCO, Carlsbad, CA, USA) for 7 days at 37°C in a humidified 5% CO_2_ atmosphere. The medium was changed every 3 days. After allowing the adherent cells to grow to approximately 80% confluence, they were detached from the tissue culture plate using 0.25% trypsin- 1 mM ethylenediamine tetraacetic acid (EDTA) solution (Sigma-Aldrich Corporation), counted, and either replated for monolayer cultures or used for spheroid formation.

### Mesenchymal stem cell spheroid formation

Dissociated rMSC monolayers (Passage 3) were resuspended in medium to obtain a single cell suspension. MSCs (8 × 10^5^ cells/ml, corresponding to approximately 100, 1,000, and 10,000 cells/well) were added to Nunc®Low Cell Binding Surface 96-well plates (Thermo Scientific Nunc A/S, Roskilde, Denmark) and incubated in DMEM medium supplemented with FBS and PS at 37°C for up to 7 days.

### Osteogenic induction in mesenchymal stem cell spheroids

To generate osteogenic differentiation, MSC spheroids were cultured with DMEM in the presence of OIR (Takara Bio Inc., Otsu, Japan), which included ascorbic acid, hydrocortisone, and β-glycerophosphate. Culture of MSC spheroid was allowed to grow for 7 days with media exchanged every 2 days. For immunocytochemistry and, ALP and AR staining, MSC spheroids incubated with OIR were removed from the well and plated onto 13-mm cover glass for 2 hours. Parental rMSCs were seeded on to 13-mm cover glasses and cultured with DMEM containing 10% (v/v) FBS and an OIR for 7 days.

### Real-time reverse transcription-polymerase chain reaction

The total RNA was isolated from differentiated spheroids or monolayer MSCs cultured with the OIR for 7 days using a High Pure RNA Isolation Kit (Roche Diagnostics, Basel, Switzerland), according to the manufacturer’s instructions. Total RNA (1 μg) was transcribed into cDNA using random, oligo (dT) primers, and reverse transcriptase in a total volume of 10 μl (ReverTra Ace® qPCR RT Kit; Toyobo Co., Ltd., Osaka, Japan). Reverse transcription was performed at 37°C for 15 min, at 50°C for 5 min, and then at 98°C for 5 min. The resulting templates were amplified in a LightCycler® Nano RT-PCR system (Roche Diagnostics) according to the manufacturer’s protocol. *G3PDH* was used as an internal control. Relative mRNA expression was determined as the ratio of *RUNX-2, OSX*, *OPN*, or *BSP* mRNA to *G3PDH* mRNA. The sequence of the specific primers and Universal ProbeLibrary (Roche Diagnostics) probe numbers used for the quantitative RT-PCR analysis are listed in Table 1. All reactions were run in triplicate. Results are expressed as fold-increases in mRNA expression (normalized to that of *G3PDH* mRNA) and compared with results for monolayer rMSCs treated with an OIR on Day 7.

**Table 1:** **Primers and probes used in this study**

**Gene**	**Forward and reverse primers (5′ → 3′)**	**Probe no.**
G3PDH	AATGTATCCGTTGTGGATCTGA	80
	GCTTCACCACCTTCTTGATGT	
RUNX-2	GGCCCTGGTGTTTAAATGG	83
	AGCACTCACTGACTCGGTTG	
OSX	CCCAACTGTCAGGAGCTAGAG	26
	GATGTGGCGGCTGTGAAT	
OPN	GGCTACAGCATCTGAGTGTTTG	82
	CGGTGAAAGTGGCTGAGTTT	
BSP	TCGGAAGAAAATGGAGATGG	63
	TTCCTCTTCATTTGAAGTCTCCTC	
ALP	ACGAGGTCACGTCCATCCT	89
	CCGAGTGGTGGTCACGAT	

G3PDH, *glyceraldehyde 3-phosphate dehydrogenase*; RUNX-2, *runt-related transcription factor-2*; OSX, *osterix*; OPN, *osteopontin*; BSP, *bone sialoprotein*; ALP, *alkaline phosphatase.*

### Immunocytochemical detection of osterix in mesenchymal stem cell spheroids

After culturing for the indicated time, spheroids and parental cells on 13-mm cover glass were fixed with 4% paraformaldehyde for 10 min and then washed in 0.1% Triton X-100 in phosphate-buffered saline (PBS) for 15 min. They were incubated with a rabbit polyclonal antibody to Sp7/OSX (1:100; Abcam, Cambridge, UK), at 4°C overnight. After washing with PBS, both spheroids and cells were incubated in a mixture of anti-rabbit Immunoglobulin G antibody conjugated with Alexa Fluor® 568 (1:200; Molecular Probes, Eugene, OR, USA). To visualize the nuclei, immunostained spheroids and cells were counterstained with 4, 6-diamidino-2-phenylindole (DAPI; Vector Laboratories, Inc., Burlingame, CA, USA).

### Alkaline phosphatase and Alizarin red staining

ALP and AR staining were assessed in both MSC spheroids and monolayer rMSCs induced after exposure to the OIR for 7 days. ALP staining was performed using an ALP staining kit (Takara Bio Inc., Otsu, Japan), according to the manufacturer’s instructions. Calcified deposits were detected by AR staining. Both MSC spheroids and monolayer rMSCs were fixed for 10 min in 4% paraformaldehyde in PBS and was rinsed twice with distilled water. The cells were stained at room temperature for 5 min with AR.

### The rat calvarial bone defect model

Ten-week-old male Fischer rats (weight approximately 250 g; *n* = 20) were anesthetized with 2% isoflurane (Abbott Laboratories, Abbott Park, IL, USA) and an air mixture gas flow of 1 l/min using an anesthesia gas machine (Anesthesia Machine SF-B01; MR Technology, Inc., Tsukuba, Japan). After shaving the skin, an incision was made in the skull, and the periosteum was opened, exposing the surface of the calvarial bones. A circular bone defect (full-thickness, 5 mm diameter) was created in the left parietal bone with a trephine drill and was irrigated with saline to remove bone debris. MSC spheroids were then implanted into the defects. Five MSC spheroids or monolayer rMSCs (5 × 10^4^ cells), which had been cultured in medium containing the OIR for 7 days, were loaded into 20 μl Matrigel™ on ice and transplanted into the defects at room temperature. Controls for the transplant experiments included calvarial defects implanted with or without Matrigel™. Our animal experimentation protocols were approved by the Animal Care and Use Committee of Fukuoka Dental College, Fukuoka, Japan (No. 13006).

### Evaluation of bone regeneration

Bone regeneration was evaluated using an *in-vivo* micro-CT system (Skyscan-1176 Micro-CT Scanner; Burker MicroCT, Kontich, Belgium) at 50 kVp and 500 μA on rats under anesthesia (as described above), 4 weeks after implantation. Each image data set consisted of a scan size of approximately 35 mm. The percentage of newly formed bone in each calvarial bone defect was calculated as previously described [28,29].

After micro-CT scanning, rats were sacrificed by injecting an overdose of isoflurane. Subsequently, the cranial tissues containing MSC spheroids, monolayer rMSCs, or control substances were immediately excised. Tissue specimens were fixed in 4% paraformaldehyde in PBS, decalcified in 10% EDTA for 4 weeks at 4°C, and then was embedded in paraffin. Paraffin sections were then stained with hematoxylin and eosin and Azan Mallory to visualize any histological changes. In histomorphometric analysis, the percentage of the volume of the newly formed bone per the original volume of the defect was calculated.

### Statistical analysis

The results are presented as means ± standard deviation. Group results were compared using one-way analysis of variance and Scheffe’s multiple comparison tests. A p-value of <0.05 was considered statistically significant.
